# Different regulatory mechanisms of the capsule in hypervirulent *Klebsiella pneumonia*: “direct” wcaJ variation vs. “indirect” rmpA regulation

**DOI:** 10.3389/fcimb.2023.1108818

**Published:** 2023-04-25

**Authors:** Weiwen Wang, Dongxing Tian, Dakang Hu, Wenjie Chen, Ying Zhou, Xiaofei Jiang

**Affiliations:** ^1^ Department of Clinical Laboratory, Huashan Hospital of Fudan University, Shanghai, China; ^2^ Department of Laboratory Medicine, Affiliated Hospital of Jining Medical University, Jining, China; ^3^ Department of Laboratory Medicine, Taizhou Municipal Hospital, Taizhou, China; ^4^ Department of Infectious Disease, Huashan Hospital of Fudan University, Shanghai, China; ^5^ Department of Clinical Laboratory, Shanghai Pulmonary Hospital of Tongji University, Shanghai, China

**Keywords:** capsule, *wcaJ*, *rmpA*, Virulence, hypervirulent *Klebsiella pneumoniae*

## Abstract

**Introduction:**

Hypervirulent *Klebsiella pneumoniae* produce an increased amount of capsular substance and are associated with a hypermucoviscous phenotype. Capsule production is regulated by capsular regulatory genes and capsular gene cluster variations. In the present study, we focus on the effect of *rmpA* and *wcaJ*on capsule biosynthesis.

**Methods:**

Phylogenetic trees were constructed to analyze wcaJ and rmpA sequence diversity in different serotypes hypervirulent strains. Then mutant strains (K2044^ΔwcaJ^, K2044^K1wcaJ^, K2044^K2wcaJ^and K2044^K64wcaJ^) were used to verify the effects of wcaJ and its diversity on capsule synthesis and strain virulence. Furthmore, the role of rmpA in capsular synthesis and its mechanisms were detected in K2044^ΔrmpA^ strain.

**Results:**

RmpA sequences are conversed in different serotypes. And rmpA promoted the production of hypercapsules by simultaneously acting on three promoters in cps cluster. Whereas w*caJ*, its sequences are different in different serotypes, and its loss result in the termination of capsular synthesis. Moreover, the results verified that K2 *wcaJ* could form hypercapsule in K2044 strains (K1 serotype), but K64 w*caJ* could not.

**Discussion:**

The interaction of multiple factors is involved in capsule synthesis, including w*caJ* and r*mpA*. RmpA, an known conserved capsular regulator gene, acts on cps cluster promoters to promote the production of the hypercapsule. WcaJ as initiating enzyme of CPS biosynthesis, its presence determines the synthesis of capsule. Besides, different from rmpA, w*caJ* sequence consistency is limited to the same serotype, which cause wcaJ functioning in different serotype strains with sequence recognition specificity.

## Introduction

Hypervirulent *Klebsiella pneumoniae* (hvKp) has recently elicited concern because of its ability to cause serious invasive disease, such as liver abscesses, bacteremia, and pleural empyema (Russo and Marr, 2019; Chen et al., 2021). Unlike classic *K. pneumoniae* strains, hypervirulent strains typically possess a thick, hypermucoid capsule (Paczosa and Mecsas, 2016). The capsule is a key virulence factor that renders hvKp resistant to the complement system and to phagocytosis (Xu et al., 2021). However, the mechanisms underlying capsule production and pathogenicity remain unclear.

To date, studies have demonstrated that capsule synthesis is affected by many different regulatory elements, such as RmpA/RmpA2, KvrA/KvrB, CRP, and RcsAB (Walker and Miller, 2020). Among these, *rmpA/A2* is a major regulator of the mucoid phenotype of *K. pneumoniae*. Studies have shown that both the salmochelin (*iroBCDN*)/aerobactin (*iucABCDiutA*) systems and *rmpA/A2* are found on the large plasmid of almost all reported hvKp strains (Struve et al., 2015). However, it is unknown how *rmpA/A2* regulates the synthesis of capsules in hvKp strains.

The genes needed for capsule synthesis are located on a chromosomal operon, *cps.* Approximately 110 types of capsule have been defined in *Klebsiella* spp.; each has a distinct capsular polysaccharide (CPS; also called K antigen) chemical structure (Follador et al., 2016). Nevertheless, the most common capsule locus associated with hvKp is K1, followed by K2 (Fung et al., 2002; Lee et al., 2016). Moreover, based on extensive results from mouse experiments, K1 and K2 strains are generally more virulent than strains of other serotypes (Mizuta et al., 1983; Yu et al., 2008), suggesting that distinct capsules can lead to differences in bacterial virulence.

The *cps* cluster locus comprises three main components: at the 5’ end, six relatively conserved genes (*galF, orf2, wzi, wza, wzb*, and *wzc*) are responsible for the transportation and processing of CPSs; the highly variable middle region regulates the assembly and polymerization of the CPSs; and the 3’ end region mainly determines the synthesis of monosaccharides, with all these sequences being highly conserved (Shu et al., 2009). Furthermore, the capsules of *K. pneumoniae* are synthesized *via* a *wzy*-dependent pathway. Generally, the synthesis of the capsular repeat is initiated by the initial glycosyltransferase (GT)–WbaP or WcaJ (Whitfield, 2006) and further catalyzed by specific (non-initial) GTs, allowing the addition of sugars (Whitfield, 2006). The lipid-linked repeat units are flipped across the plasma membrane by Wzx and then polymerized by Wzy (Whitfield and Roberts, 1999). Subsequently, the channel Wza, together with regulators Wzb and Wzc, which control the process of polymerization and transportation, exports the polymer to the surface of the bacterium (Shu et al., 2009), where it is anchored to the cell surface by Wzi (an outer-membrane lectin) (Whitfield and Paiment, 2003; Bushell et al., 2013).

In conclusion, capsule synthesis is not only affected by regulatory factors, but is also associated with diversity among its constituent genes. In the present study, to develop a better understanding of the process of capsule synthesis in hvKp strains, we aimed to analyze the effects of the initial glycosyltransferase gene in capsule synthesis (wcaJ* and of the capsular regulatory gene (*rmpA*) on capsule production.

## Methods

### Strains and definitions

All 188 sequenced strains of hypervirulent *K. pneumoniae* were available in the Genbank database (as of May 2022), and the characteristics of these genomes are listed in **Table S1**. Additionally, 530 K*. pneumoniae* clinical isolates were previously collected from nine hospitals in seven provinces between January 2017 and February 2018. We selected strains of the three main hypervirulent serotypes (K1, K2, and K64) from these *K. pneumoniae* isolates, for a total of 48 strains; the details of these strains are also provided in **Table S1**. MLST analysis was conducted using the Pasteur Institute MLST website for *K. pneumoniae* (http://bigsdb.pasteur.fr/klebsiella/klebsiella.html). Additionally, K serotypes were determined using Kaptive 2.0 (https://github.com/katholt/kaptive). Virulence genes (iro and iuc*;* rmpA and rmpA2) were identified using the blastn tool (https://blast.ncbi.nlm.nih.gov/). Isolates were classified as the hvKp genotype if they contained *iuc* and/or *iro*, and *rmpA* and/or *rmpA2*.

### Gene sequencing and phylogenetic analysis

PCR and sequencing were carried out to screen for the *wcaJ/wbaP* gene in isolates obtained from the Genbank dataset and from clinical sources. The primers employed are shown in **Table S2**. The *wcaJ/wbaP* sequences were aligned using the Clustal W software package, and maximum-likelihood phylogenetic trees were constructed using the MEGA v7.0.26 software package with 1000 bootstrap values.

### Construction of plasmids and gene knockout strains

Plasmids pACYC-wcaJ^K1^, pACYC-wcaJ^K2^, and pACYC-wcaJ^K64^ were constructed using the NEBuilder HiFi DNA Assembly Cloning Kit (NEB), following the manufacturer’s recommended protocol. Additionally, the pACYC184 plasmid was electrotransferred into NTUH-K2044 as a negative control.

The gene knockout strains K2044^ΔwcaJ^ and K2044^ΔrmpA^ were constructed using the λ-Red homologous recombination system, as previously reported (Datsenko and Wanner, 2000). The primer sequences employed are presented in **Table S2**. When necessary, appropriate antibiotics were added at the following final concentrations: chloramphenicol (chlo): 60 μg/mL for pACYC184; kanamycin (kana): 100 μg/mL for K2044^ΔwcaJ^ and K2044^ΔrmpA^.

### Growth conditions

The effect of wcaJ on the growth of *K. pneumoniae* was measured. In brief, overnight cultures of all *K. pneumoniae* strains were diluted at 1:100 in broth supplemented with different appropriate antibiotics. The cultures were incubated at 37°C and A_600_ was measured every 30 min.

### Transmission electron microscopy

*K. pneumoniae* bacteria in the mid-log growth phase were collected and fixed with 2.5% glutaraldehyde overnight at 4°C, then washed twice in 0.1 M phosphate buffer. Next, bacteria were post-fixed with 1% osmium tetroxide for 1 h, washed twice in 0.1 M phosphate buffer water, and washed twice in water. The samples underwent subsequent dehydration in increasing grades of alcohol (30%, 50%, 70%, 80%, 95%: 15 minutes; 100%: 2 × 10 min). Next, infiltration and embedding were performed using Spurr’s resin and samples were polymerized at 60°C for 48 h. Samples were examined using a Tecnai G2 Spirit Twin transmission electron microscope and corresponding images were recorded.

### Mucoviscosity assay and capsule quantification

The mucoviscosity of the Klebsiella strains was measured as previously described (Bachman et al., 2015). An overnight culture grown in LB was diluted at 1:100 in media and grown at 37°C. At 6 h, the culture was centrifuged at 1000 × g for 5 min, followed by immediate measurement of the OD_600_ of the supernatant.

Uronic acid was extracted and quantified as described previously (Mike et al., 2021); the test strains were cultured for 6 h. Subsequently, 500 µL culture was mixed with 100 µL of 1% Zwittergent 3–12 detergent and heated for 20 min at 50°C, then centrifuged for 5 min at 13,000 × g. Next, 300 µl of supernatant was mixed with 1.2 mL absolute ethanol and centrifuged for 5 min at 13,000 × g. The pellet was dried and resuspended in 200 µl of sterile water, to which 1.2 mL of tetraborate solution (12.5 mM sodium tetraborate in sulfuric acid) was added. This was incubated for 5 min at 100°C, followed by immediate cooling on ice for at least 10 min, which was then followed by addition of 20µl of hydroxyphenyl reagent. After 5 min incubation at room temperature, OD was determined at 520 nm.

### Biofilm formation

Biofilm assay was performed, following previously published methods, in 96-well plates. Briefly, 200 μL of mid-log phase bacteria cells (1.5x10^7^CFU/ml) was added to a 96-well plate and incubated at 37°C for 24 h. All cultures were removed and the wells were washed twice with phosphate-buffered saline (PBS), then stained with 200 μL of 0.1% crystal violet for 20 min and rinsed twice with PBS. Stained biofilms were solubilized with 95% ethanol and quantified by measuring the OD_595_ value.

### Phagocytosis and invasion assays

RAW 264.7 murine macrophages were grown in DMEM medium supplemented with 10% FBS. Macrophages (1x10^5^/well) were seeded into 24-well plates and infected at a MOI of 50 (bacteria/cell). Subsequently, plates were centrifuged at 200 × g for 5 min and incubated at 37°C with 5% CO_2_. After 1.5 h, cells were washed three times with PBS. Next, cells were further incubated for 1.5 h with meropenem to kill extracellular bacteria. Cells were then rinsed again three times with PBS and lysed with 0.2% Triton X-100 for 20 min. Serial dilutions of the lysate were plated for LB agar plates to determine the number of CFUs per unit volume. Three biological replicates per strain were used per experiment.

Mid-log-phase *K*. pneumoniae in DMEM medium containing 10% FBS were added to the wells (at MOI 50), incubated for 2 h, and washed three times with PBS. Cells were then incubated for 2 h with fresh medium containing meropenem. Finally, the number of intracellular bacteria was determined by the number of CFUs on the LB agar.

### Serum resistance assay

For serum survival assay, the mid-log phase bacteria cells (1.5x10^6^ CFU/ml) were mixed with normal human serum at a 1:3 ratio and incubated at 37°C for 2 h. Bacteria were grown overnight in LB for enumeration of viable bacteria.

### Infection model of *Galleria mellonella* larvae

G. mellonella killing assays were carried out on the six isolates. Ten larvae weighing approximately 300 mg were randomly selected for each strain. For each of these, 10 μl of bacterial suspension (1×10^6^ CFU/ml) in PBS or 10µl of PBS (control group) was injected into the last left proleg. The insects were incubated at 37°C in the dark and observed for 96 h. Larvae were considered dead when they repeatedly failed to respond to physical stimuli.

### Quantitative RT-PCR

Total RNA was extracted from the bacteria in the logarithmic growth phase. Following this, the RNA was converted to cDNA using the PrimeScript™ RT Reagent Kit. Real-time PCR was performed using a LightCycler^®^ System. Relative gene expression levels were measured in terms of Ct values and analyzed using the 2^-ΔΔCt^ method. The primer sequences are presented in **Table S3**. RpoB was used as an internal control. Three biological replicates per strain were used.

### Statistical analysis

All experiments were performed with biological replicates, and graphical and numerical data analysis was carried out using Graphpad Prism 9.0. Analyses included two-tailed Student’s t-tests, one-way analysis of variance (ANOVA), two-way ANOVA, and log-rank tests. Statistical significance was defined as *p* < 0.05.

## Results

### The distribution of *wcaJ/wbaP* in hvKp

**Figure 1:**
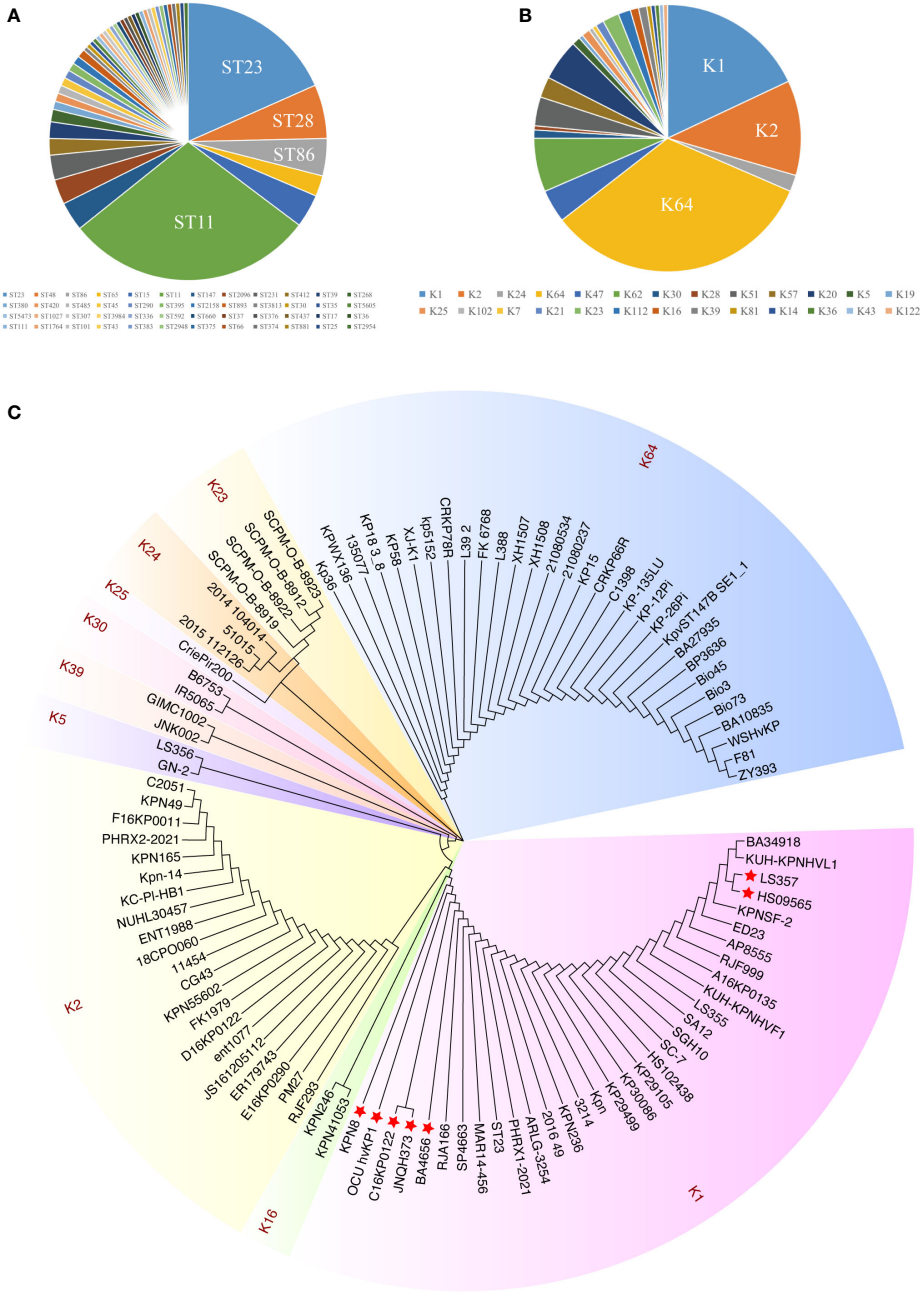
The distribution and sequence diversity of *wcaJ/wbaP* among hvKp strains. **(A)** MLST of the 226 completely sequenced hvKp strains available from GenBank. **(B)** The serotypes of the 226 hvKp strains available from GenBank. **(C)** A *Klebsiella pneumoniae* phylogenetic tree of *wcaJ* sequences in various *K. pneumoniae* serotypes. The tree was constructed using the maximum likelihood method with 1,000 bootstrap replicates in the MEGA7 software package. Different colors represent different serotypes. The strains marked with red star refer to nucleotide mutations in its wcaJ sequence.

Multilocus sequence typing (MLST) of 226 hvKp strains showed that ST11 was the most prevalent sequence type (ST), followed by ST23, ST48, and ST86 (**Figure 1A**). Additionally, the most prevalent capsule types were the K64, K1, and K2 serotypes (**Figure 1B**). These results are consistent with previous findings. Previously, hvKP strains have usually been found to be associated with sequence types ST23, ST65, and ST86 and with serotype K1/K2. However, in recent years, virulence plasmids have been acquired in CRKP strains to an increasing extent (ST11), creating hypervirulent and carbapenem-resistant *K. pneumoniae* (CR-hvKP) and resulting in widespread epidemics (Lan et al., 2021; Tian et al., 2022).

We also analyzed the expression of *wcaJ/wbap* in different serotypes. Considering the transfer of Gal-1-P from UDP-galactose or UDP-glucose to und-P (Whitfield, 2006), we analyzed *wcaJ* and *wbap* sequences by constructing evolutionary trees separately. Firstly, as shown in **Figure 1C**, *wcaJ* was present in the K1, K2, K5, K16, K23, K24, K25, K30, K39, and K64 serotypes, whereas *wbaP* was present in the K19, K20, K21, K43, K47, K51, K57, K62, and K112 strains (**Figure S1**). The results showed that *wcaJ* and *wbaP* sequences of the same serotype were clustered in the same branch, indicating that wcaJ or *wbaP* sequences in the same serotype are relatively conserved. However, different nucleotide mutations also appeared in the wcaJ sequence of the same serotype, such as the K1 serotype (**Figure S2**).

### The diversity of *wcaJ/wbaP* sequences in clinical isolates

**Figure 2:**
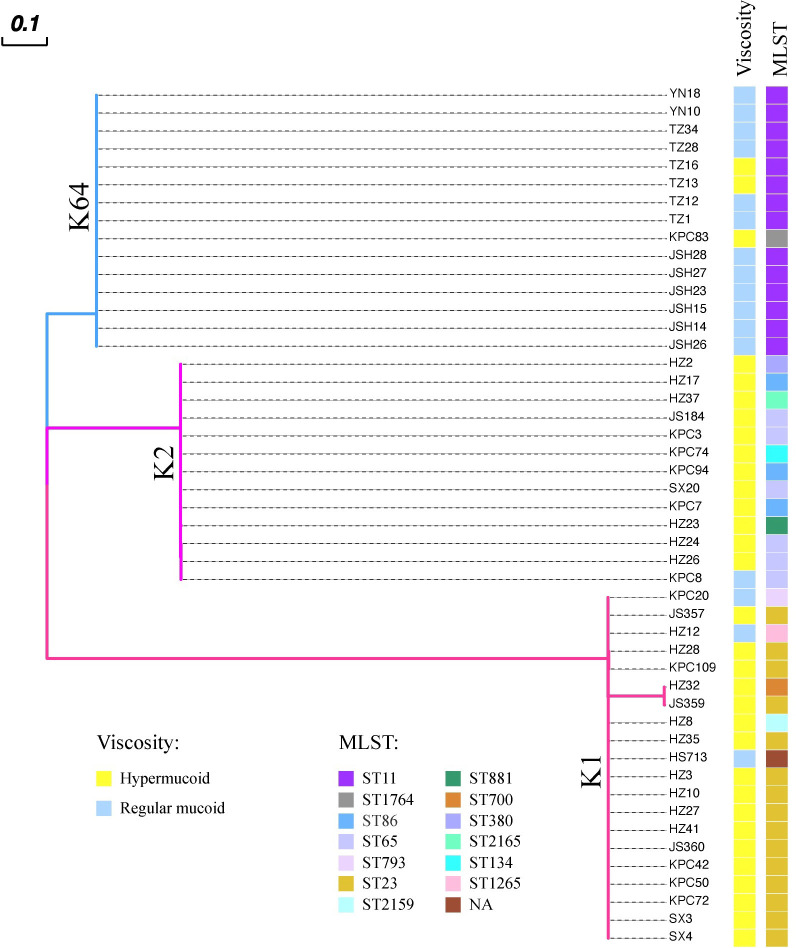
The distribution of *wcaJ* among distinct *Klebsiella pneumoniae* serotypes (K1, K2, and K64) in 48 clinical hvKp isolates. Each color branch represents a different serotype. The *wcaJ* sequences within the same serotype are identical. The MLSTs are displayed for different strains; presence of hypermucoid (> 5 mm) or normal capsules was determined using string tests.

To date, two main categories of hvKp serotype appear to have been identified: 1) K1 and K2; and 2) K64. Therefore, we next focused on studying the *wcaJ* sequences in the three common hvKp serotypes: K1, K2, and K64. To this end, a total of 48 hvKp isolates were collected from six hospitals, including K1 (n = 20), K2 (n = 13), and K64 (n=15). The results, as presented in **Figure 2**, showed that the *wcaJ* sequences were identical within strains belonging to the same serotype (including K1, K2, and K64 clinical strains). Furthermore, 75% of the K1 serotype was composed of ST23, while the K2 serotype comprised a greater variety of STs, such as ST65, ST86, and ST25. Finally, the K64 serotypes were mostly composed of ST11. Interestingly, a majority of the K1/K2 strains were hypermucoid, whereas most of the K64 strains were not. It is unknown whether different *wcaJ* sequences play an important role in hypercapsule formation.

### Capsule formation in different recombinant *K. pneumoniae* strains

**Figure 3:**
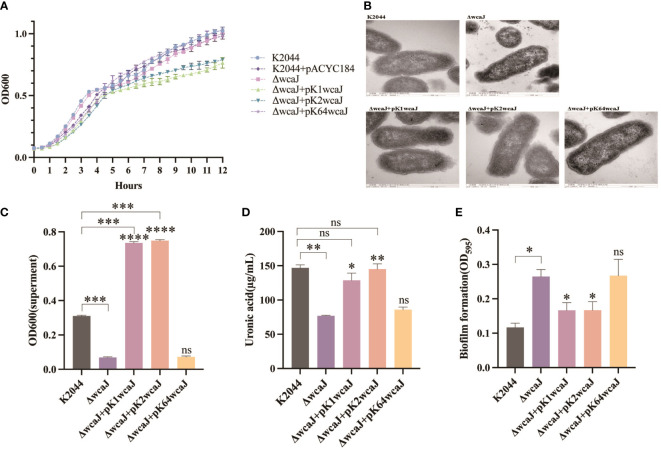
Capsule formation in various *Klebsiella pneumoniae* mutants. **(A)** The 12 h growth curves of different strains. **(B)** Transmission electron microscopy images of the capsule. Assessment of **(C)** the mucoviscosity of the culture supernatants, **(D)** the production of uronic acid, and **(E)** the formation of biofilm. K2044^ΔwcaJ^ was used as a negative control. An unpaired two-sided Student’s t-test was used for comparisons between K2044^ΔwcaJ^ and its mutants. Each data point represents an average of three independent replicates (n = 3). The values of culture supernatants, uronic acid, and biofilm formation were normalized to OD600. Data are presented in the form mean ± SEM. ***P < 0.001, **P < 0.01, *P < 0.05, ns: not significant. ****P < 0.0001, ***P < 0.001, **P < 0.01, *P < 0.05, ns, not significant.

The effect of *wcaJ* on capsule formation was analyzed by constructing various mutants: K2044^K1wcaJ^, K2044^K2wcaJ^, and K2044^K64wcaJ^. We found that the K2044^K1wcaJ^ and K2044^K2wcaJ^ recombinants exhibited significantly slower rates of growth compared to other mutants (**Figure 3A**). However, the presence of pACYC184 plasmid did not affect the growth of the strain. The growth defects in K2044^K1wcaJ^ and K2044^K2wcaJ^ require further exploration. Next, electron microscopy results showed that K2044, K2044^K1wcaJ^, and K2044^K2wcaJ^ (but not K2044^ΔwcaJ^ or K2044^K64wcaJ^) produced a loose and thick mucus substance (**Figure 3B**), suggesting that the K2044^K64wcaJ^ mutants had impaired capsule formation abilities, similar to those of the K2044^ΔwcaJ^ mutants.

Viscosity and uronic acid were subsequently measured. The results indicated that knockout of *wcaJ* significantly reduced capsular viscosity, and the complement of p-K1*wcaJ* or p-K2*wcaJ* caused hypercapsulation; these were even more hypermucoid than the K2044 parent strains (**Figure 3C**). In addition, capsule formation was quantified by measuring uronic acid. As shown in **Figure 3D**, decreased uronic acid was observed in K2044^ΔwcaJ^, but K2044^K1wcaJ^ and K2044^K2wcaJ^ produced uronic acid to an extent similar to K2044. The biofilm, an important bacterial protective mechanism, is another substance existing on the surface of cells. There was an inverse correlation between capsule production and biofilm formation (**Figure 3E**), as the K2044^ΔwcaJ^ and K2044^K64wcaJ^ capsule-null mutants were better able to form biofilms than K2044, K2044^K1wcaJ^, or K2044^K2wcaJ^. Furthermore, there was no significant difference between K2044^ΔwcaJ^ and K2044^K64wcaJ^ in terms of capsular viscosity, uronic acid, or biofilm production.

### Differences in CPS synthesis clusters and *wcaJ* sequences

**Figure 4:**
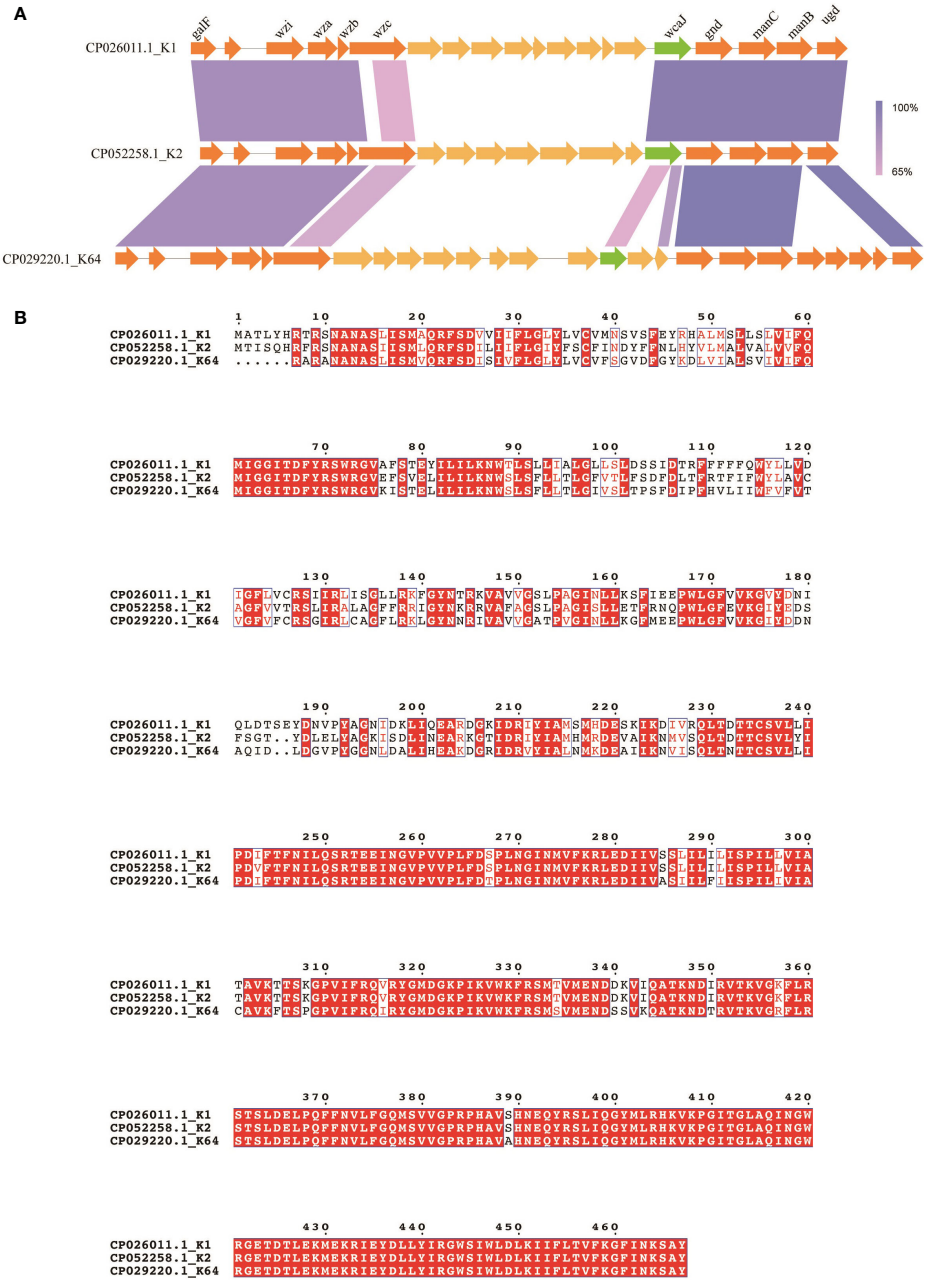
Comparison of *wcaJ* sequences among K1, K2, and K64 serotypes. **(A)** The genetic differences between the *cps* clusters of the K1, K2, and K64 serotypes. **(B)** The WcaJ protein sequences in the K1, K2, and K64 serotypes. Alignment was performed using the MUSCLE sequence alignment tool.

To summarize, the plasmids carrying K64 *wcaJ* exhibited impaired capsule formation, whereas the complement of K2 *wcaJ* did not. The associated molecular mechanism was unclear. A comparison was made of the composition of *cps* loci among K1, K2, and K64 serotypes. The results revealed the presence of conserved genes within the three serotypes, including *galF, wzi, wza, wzb, wzc, wcaJ, gnd, manC, manB*, and *ugd* (**Figure 4A**). Additionally, wcaJ protein alignment showed that F244-Y467 were identical in K1 and K2, but not in the K64 serotype (**Figure 4B**).

### Cell invasion and phagocytosis of different recombinants

**Figure 5:**
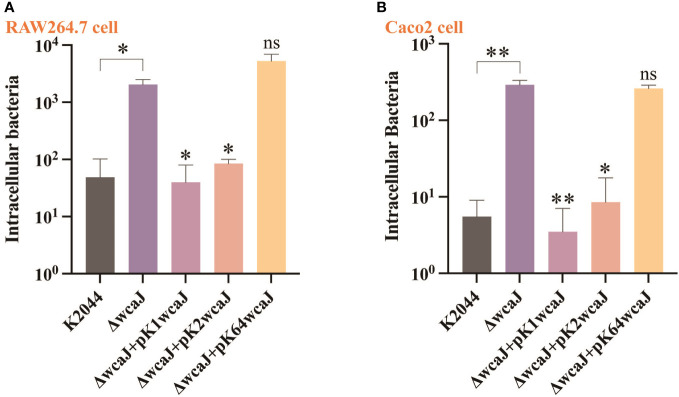
The cell internalization phenotypes of different *Klebsiella pneumoniae* mutants. The invasion assay was performed in **(A)** macrophage RAW 264.7 cells and **(B)** intestinal epithelial Caco2 cells. The K2044^ΔwcaJ^ strain was taken as a control group. Data were analyzed via one-way ANOVA. Each data point represents three replicates. **P < 0.01, *P < 0.05, ns: not significant.

Previous studies have demonstrated that the hypercapsule of hvKp is essential for resistance to phagocytosis. Compared with K2044^ΔwcaJ^, the bacterial invasion of RAW264.7 and Caco2 cell lines was equally significantly suppressed in the K2044, K2044^K1wcaJ^, and K2044^K2wcaJ^ groups. However, the number of bacterial invasions did not differ between the K2044^ΔwcaJ^ and K2044^K64wcaJ^ groups (**Figure 5**). The above results indicate that the presence of the capsule reduced the invasion and phagocytosis of hvKp strains.

### The effects of *wcaJ* on *K. pneumoniae* virulence

**Figure 6:**
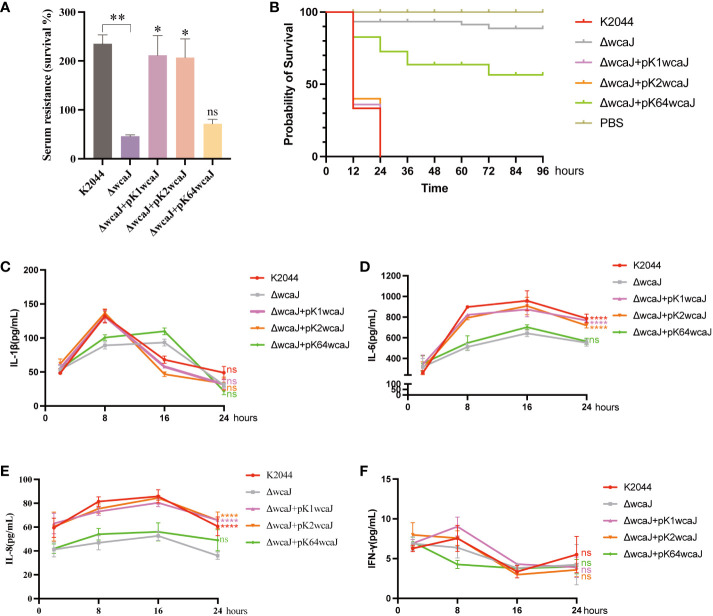
The virulence phenotype of different *Klebsiella pneumoniae* mutants. **(A)** Serum resistance of K2044, K2044^K1wcaJ^, K2044^K2wcaJ^, and K2044^K64wcaJ^, with K2044^ΔwcaJ^ as a reference. **(B)** The survival curves of infected *Galleria mellonella* larvae; a log-rank (Mantel–Cox) test was used to analyze the survival curves. **(C-F)** Production of the inflammatory cytokines **(C)** IL-1β, **(D)** IL-6, **(E)** IL-8, and **(F)** IFN-γ was measured by ELISA at 2, 8, 16, and 24 h. The K2044^ΔwcaJ^ mutant was analyzed as negative control, and two-way ANOVA tests were performed. Data are presented in the form mean ± SEM. ****P < 0.0001, ***P < 0.001, **P < 0.01, *P < 0.05, ns, not significant.

Since the capsule acts as an important virulence factor, we wondered whether wcaJ sequence variations could affect the virulence of hvKp isolates. As shown in **Figure 6A**, K2044, K2044^K1wcaJ^. and K2044^K2wcaJ^ exhibited significantly higher levels of serum resistance than K2044^ΔwcaJ^. As shown in **Figure 6B**, when the larvae were infected with the same bacterial inoculum, K2044^ΔwcaJ^ strains exhibited significantly decreased virulence levels compared to K2044, K2044^K1wcaJ^, and K2044^K2wcaJ^. K2044^K1wcaJ^ and K2044^K2wcaJ^ exhibited similar virulence effects to K2044, but K2044^K64wcaJ^ did not. The above results instead confirm that the synthesis of hypermucoviscous capsules significantly enhanced the virulence of a given K. pneumoniae strain.

### The inflammatory response induced by wcaJ recombinants

To examine whether wcaJ mutants induced different inflammatory responses *in vitro*, RAW 264.7 cells were treated with different recombinants and cytokine production was measured by ELISA. The results showed that release of IL-1β reached a peak at 8 h (**Figure 6C**), IL-6 at 16 h (**Figure 6D**), IL-8 at 16 h (**Figure 6E**), and IFN-γ at 8 h (**Figure 6F**). Compared to K2044^ΔwcaJ^, the K2044, K2044^K1wcaJ^, and K2044^K2wcaJ^ strains significantly promoted the production of IL-6 and IL-8, but K2044^K64^wcaJ did not. This phenomenon suggests that the presence of the capsule promoted the inflammatory response. Additionally, it is worth noting that no significant production of IL-1β or IFN-γ was observed (**Figure 6F**).

### The regulatory effect of rmpA on the capsule

Studies have demonstrated there is a strong correlation between p-rmpA/rmpA2 and hypermucoviscosity/hypervirulence. Therefore, *p-rmpA/rmpA2* is among a set of genes proposed as biomarkers for detection of hvKp strains (Russo et al., 2018). A poly-G frameshift leads to the early termination of p-rmpA2 transcription. The resulting truncated product is 99 amino acids long and lacks the C-terminal DNA-binding region. We therefore next focused on the regulatory effect of *p-rmpA* on capsule production and virulence.

**Figure 7:**
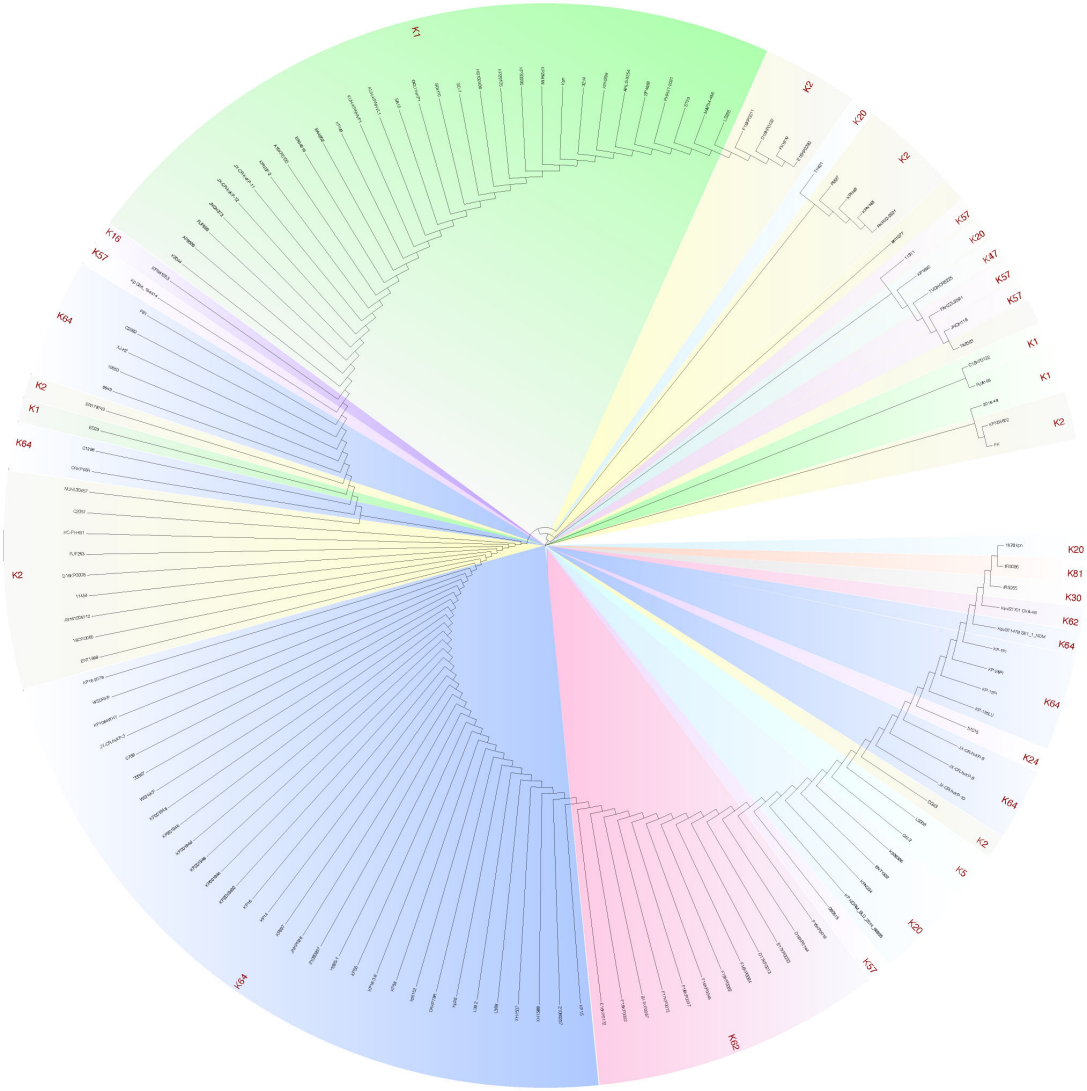
Sequence diversity of *rmpA* among hvKp strains, illustrated by the phylogenetic tree of *rmpA* sequences in various serotypes of hvKp strains. The tree was constructed using the maximum likelihood method with 1,000 bootstrap replicates in the MEGA7 software package.

**Figure 8:**
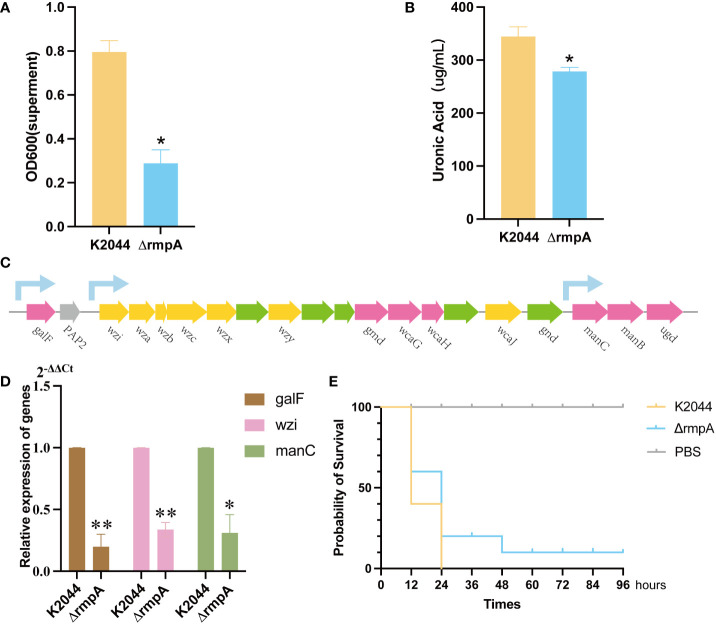
The effect of *rmpA* on the capsule and virulence of *Klebsiella pneumoniae*. **(A)** Capsule mucoviscosity. **(B)** Uronic acid production. **(C)** A diagram showing the location of *cps* clusters with the sequences of K2044 strains and the location of the relevant promoters. **(D)** qRT-PCR results for expression of *galF*, *manC*, and *wzi* in the K2044 and Δ*rmpA* strains (2^-ΔΔCt^). **(E)** The *Galleria mellonella* infection model. A log-rank (Mantel–Cox) test was used to analyze the survival curves. Data were analyzed via one-way ANOVA. **P < 0.01, *P < 0.05.

Phylogenetic tree results indicated that rmpA sequence was conserved regardless of serotype (**Figure 7**). The CPS biosynthesis capacity of the K2044^ΔrmpA^ mutant was then determined. Compared to the K2044 strains, mucoviscosity and uronic acid were markedly decreased in the K2044^ΔrmpA^ mutants (**Figures 8A, B**). The *cps* locus contains three characterized promoters, which are located upstream of *galF*, *wzi*, and *manC* (**Figure 8C**). We found that the expression of *galF, wzi*, and *manC* was decreased in the K2044^ΔrmpA^ strains (**Figure 8D**). Thus, the capsule alterations described above may have been caused by the reduced expression of genes associated with CPS biosynthesis. In consideration of the changes affecting the capsule, the virulence of K2044^ΔrmpA^ mutants was evaluated *via G. mellonella* testing. The results demonstrated that the *p-rmpA-*mediated reduction in capsule production significantly impaired the virulence of *K. pneumoniae* in *G. mellonella* (**Figure 8E**).

## Discussion

Hypervirulent K. pneumoniae strains are mainly divided into two categories: 1) common hvKp, which includes the K1 and K2 serotypes; and 2) newly-emerged carbapenem-resistant (CR)-hvKp, mainly in the form of K64 serotypes (Shon et al., 2013; Tian et al., 2022). This view is consistent with our results. The most prevalent capsule types were K64, followed by the K1 and K2 serotypes. Furthermore, the capsules in the K1/K2 serotypes were almost hypermucoviscous, but in most cases, the K64 serotype exhibited a regular capsule. Unlike non-virulent strains, hypervirulent strains produce an elevated quantity of capsular substance, thus conferring a hypermucoviscous phenotype (Chen et al., 2004; Alcántar-Curiel and Girón, 2015). However, new evidence has suggested that hypermucoviscosity and hypervirulence are two different phenotypes (Catalán-Nájera et al., 2017). Therefore, it is essential to rapidly identify the genetic determinants of hypermucoviscosity among K. pneumoniae strains.

Capsule production is transcriptionally regulated by multiple proteins. However, very little is known about how these proteins collectively control capsule production. Several studies have reported that rmpA is an important determinant of virulence for the mucoid phenotype of *K. pneumoniae* (Arakawa et al., 1991). Our studies have proven that knockdown of *rmpA* reduces mucoviscosity and CPS production, leading to a decrease in the virulence of the strain. This phenomenon is primarily mediated by the regulation of rmpA on three promoters in the *cps* clusters (including *galF, wzi*, and *manC*). Meanwhile, rmpA is a conserved capsular regulator in hvKp strains, ensuring that the synthesis of capsules can be properly regulated. However, hvKp isolates are mainly found to fall into one of several serotypes, including the K1, K2, and K64 serotypes. Furthermore, studies in mice have proven that the capsules of the K1 and K2 strains of *K. pneumoniae* are more virulent than those of many other strains (Kabha et al., 1995). It has been speculated that different gene clusters play different roles in virulence. Moreover, studies have found that the K1 and K2 strains are significantly more resistant to phagocytosis and intracellular killing by neutrophils than non-K1/K2 strains, suggesting that capsular serotype contributes to increased virulence in *K. pneumoniae* (Fung et al., 2011). Additionally, Zhang et al. demonstrated that capsule type defines the capacity of *K. pneumoniae* to evade Kupffer cells in the liver by switching between the expression of different gene clusters (Huang et al., 2022). Based on the above conclusions, diversity of the capsule plays an important role in virulence of hvKp.

Capsule biosynthesis in *K. pneumoniae* is a complex process, mediated by *cps* gene clusters. The capsules are formed *via* a *wzy*-dependent pathway. WcaJ, an initial glycosyltransferase, is responsible for the transfer of Gal-1-P from UDP-glucose to und-P, thus initiating synthesis of the capsule. Comparative analysis revealed that the *wcaJ* sequence varies among different serotypes. We constructed several mutants, including K2044^ΔwcaJ^, K2044^K1wcaJ^, K2044^K2wcaJ^, and K2044^K64wcaJ^ strains, in order to analyze the effects of *wcaJ* on capsule formation and strain virulence.K2044^ΔwcaJ^ exhibited decreased mucoviscosity and reduced uronic acid compared to K2044 strains. Meanwhile, the virulence of K2044^ΔwcaJ^ mutants was significantly reduced, and this was accompanied by a decrease in the release of inflammatory-associated factors. In addition, the disappearance of the “hypercapsule” in K2044^ΔwcaJ^ strains makes it more likely to be engulfed by phagocytes compared to K2044. However, the presence of a hypercapsule has the opposite effect in other infections. Christoph et al. have proven that hypercapsule production, which confers phagocytosis resistance, enhances dissemination and increases mortality in animal models. In contrast, mutations disrupting capsule biosynthesis impair capsule production, which enhances epithelial cell invasion, *in vitro* biofilm formation, and persistence in urinary tract infections (Ernst et al., 2020). In our experiment, hypercapsule-null K2044^ΔwcaJ^ exhibited increased biofilm formation and could invade intestinal epithelial cells easily. The results confirm that *wcaJ* is of major significance in the synthesis of capsules and the virulence of strains.

We also analyzed the effects of differences in *wcaJ* sequence on capsule synthesis. The results demonstrated that K2044^K1wcaJ^ and K2044^K2wcaJ^ formed a “hypercapsule” similar to that of K2044 isolates, but K2044^K64wcaJ^ did not. Considering that K2044 is of the K1 serotype, we focused on why K2wcaJ functions in the genetic context of K2044. Protein sequence alignment indicated that there is significantly higher similarity between K1 and K2 than between K1 and K64. In particular, the F244-Y467 protein sequences are completely consistent between the K1 and K2 serotypes, which may constitute the molecular basis for K2wcaJ to function properly. Additionally, K2044^K1wcaJ^ and K2044^K2wcaJ^ result in the same effect in terms of virulence as K2044 according to serum resistance and *G. mellonella* infection models. Meanwhile, our results also revealed that the presence of the capsule effectively induced an inflammatory response (i.e., increased IL-6 and IL-8 levels) following infection with K2044, K2044^K1wcaJ^, and K2044^K2wcaJ^ strains. IL-1β, which is produced by leukocytes, induces the production of IL-6, IL-8, and IL-1β (in a positive feedback loop), creating an unfavorable environment for infectious microorganisms (Boraschi, 2022). However, IFN-γ is not involved in the inflammatory response mediated by *K. pneumoniae* infection. Notably, the K2044^K1wcaJ^ and K2044^K2wcaJ^ strains were found to harbor growth defects compared to K2044.Additionally, taking K2044^ACYC184^ as a negative control, the introduction of pACYC184 plasmid did not affect the growth of strains. The results showed that viscosity was increased in K2044^K1wcaJ^ and K2044^K2wcaJ^ as compared to K2044, which may be the cause of growth defects. Because of the large number of copies of plasmids, K1*wcaJ* and K2wcaJ were upregulated in the presence of the plasmid, leading to increased mucoviscosity. However, there was no difference in uronic acid between K2044^K1wcaJ^/K2044^K2wcaJ^ and K2044.

Recent studies have identified two novel transcriptional regulators, RmpC and RmpD. The Δ*rmpC* strain has reduced capsule gene expression but retains the hypermucoviscous phenotype (Walker et al., 2019). Meanwhile, the Δ*rmpD* mutant is non-hypermucoviscous, but exhibits no changes in *cps* expression and produces the same amount of uronic acid (capsule) as the wild-type parental strain (Walker et al., 2020). These transcriptional regulators provide evidence that hypermucoviscosity is not dependent on capsule overproduction (uronic acid). Thus, hypermucoviscosity and CPS production should be treated as two separable phenotypic traits.

To sum up, the interactions of multiple factors are involved in capsule synthesis, including *cps* cluster loci and certain transcription factors. *RmpA*, a known capsular regulator gene, is conserved in all kinds of serotypes. RmpA could act on *cps* cluster promoters simultaneously to promote the production of the hypercapsule. In the meantime, *wcaJ* acts as an initiation glycosyltransferase gene in CPS synthesis; its knockout results in the loss of the capsule. Unlike *rmpA*, the consistency of *wcaJ* sequences is limited to within the same serotype. By constructing the *wcaJ* recombinations of different serotypes, we have discovered that K2*wcaJ* may play a role in K2044 (of the K1 serotype), but K64*wcaJ* does not. Additionally, over-expression of *wcaJ* led to an increase in viscosity, but the amount of uronic acid did not change. Previous studies have focused on the effects of nucleotide mutations in the capsular gene within the same serotype, such as *wcaJ* or *wzc* (Ernst et al., 2020; He et al., 2023). In our study, we analyzed the *wcaJ* gene in different serotypes, elucidating the mechanism underlying differences in virulence between different serotypes. Our study also has several limitations: firstly, the experiment only involved *wcaJ* sequences of three serotypes (K1, K2, and K64), and more serotypes needed to be studied; additionally, capsule synthesis involves multiple genes, such as *wza*, *wzc, wzb, wzi*, *and wzy*, and these genes also need to be compared and analyzed in different serotypes.

## Data availability statement

The original contributions presented in the study are included in the article/**Supplementary Material**. Further inquiries can be directed to the corresponding author.

## Author contributions

These authors (including WW, DT and DH) share first authorship. All authors contributed to the article and approved the submitted version.
