# Supplementary figures and images for: Different regulatory mechanisms of the capsule in hypervirulent Klebsiella pneumonia: “direct” wcaJ variation vs. “indirect” rmpA regulation
Source: Front Cell Infect Microbiol. 2023 Apr 25;13:1108818. doi: 10.3389/fcimb.2023.1108818 (PMC10168181; doi:10.3389/fcimb.2023.1108818)

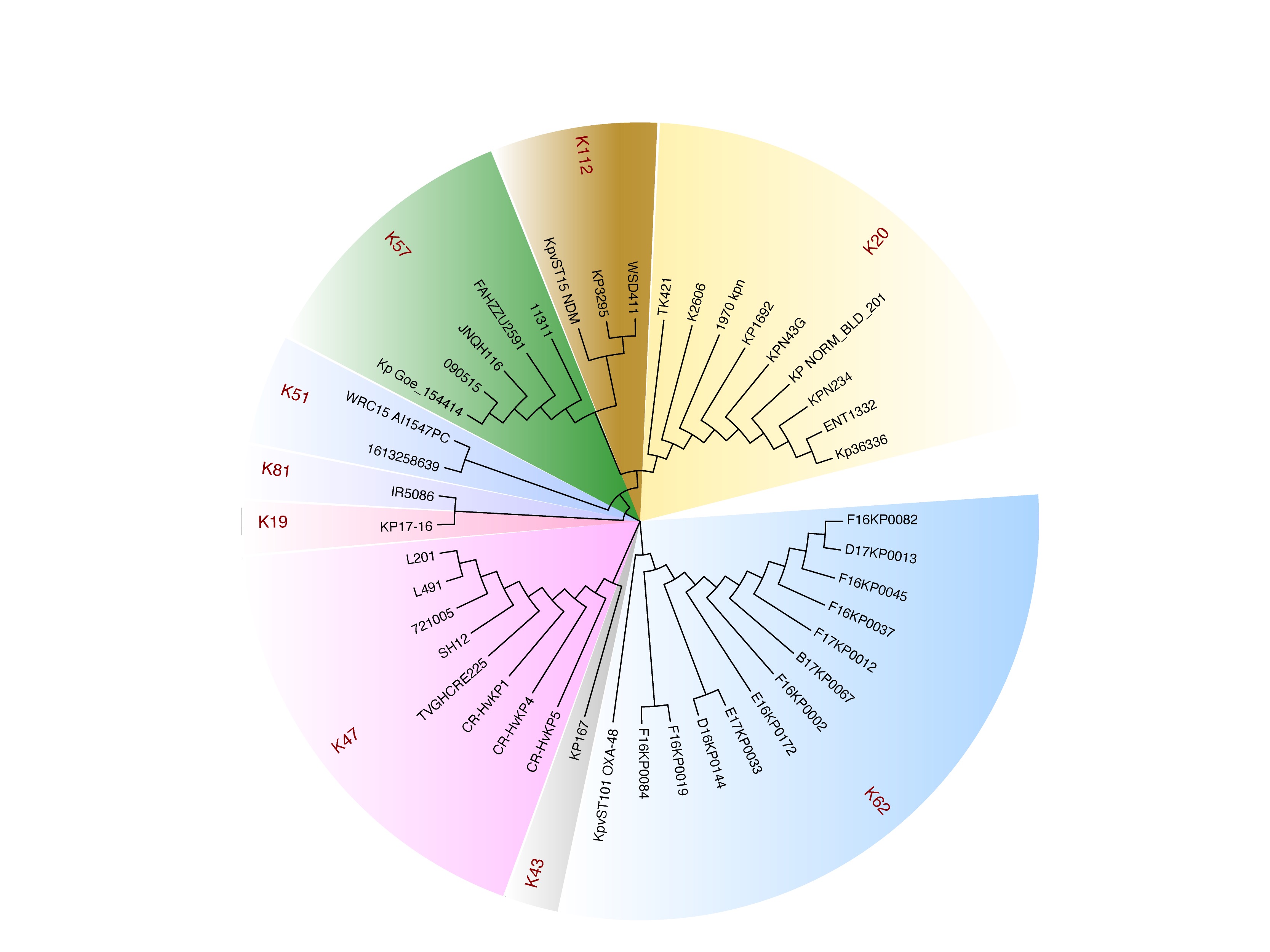

Supplement: Supplementary file 1 [file Image_1.tif]

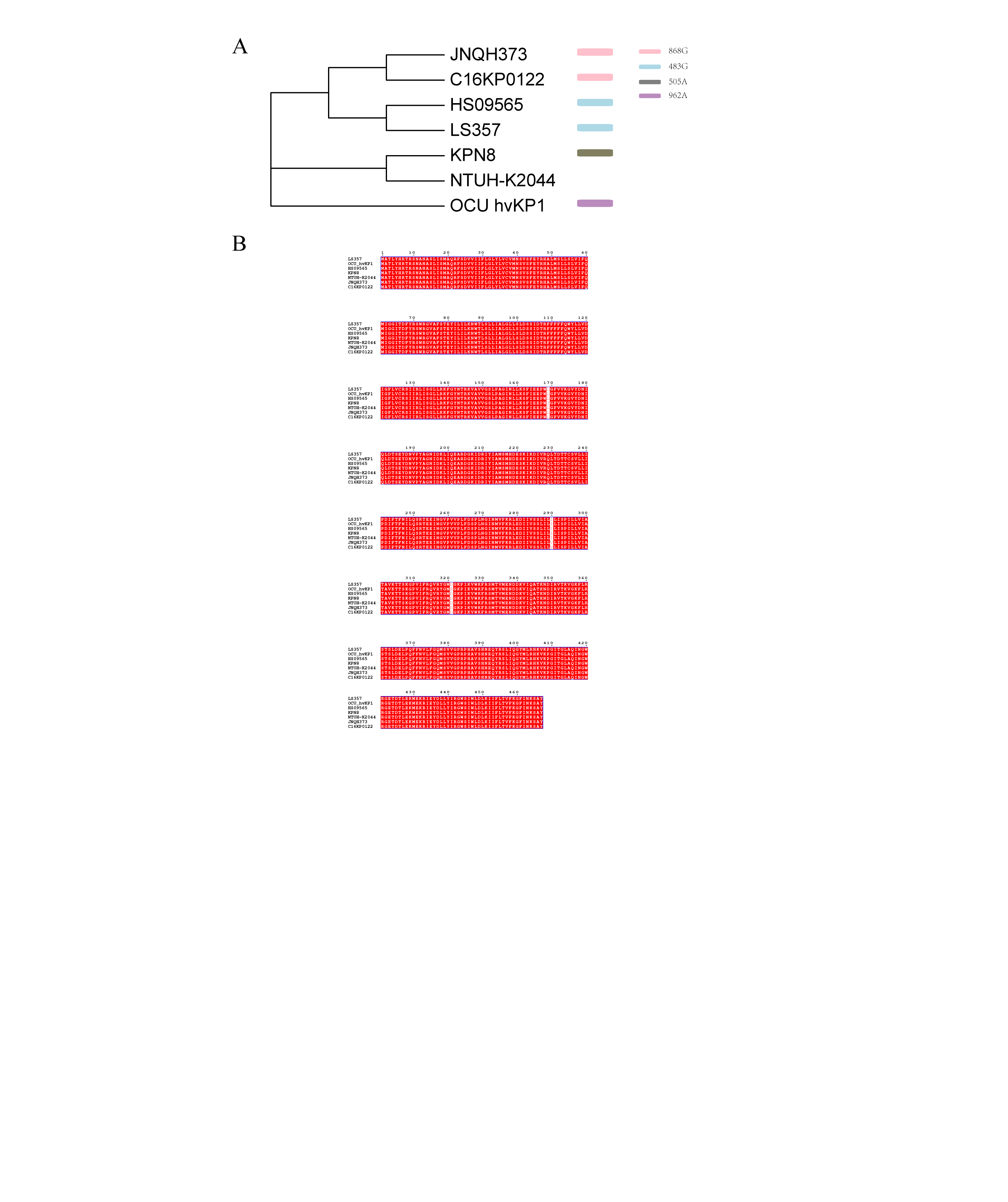

Supplement: Supplementary file 2 [file Image_2.tif]

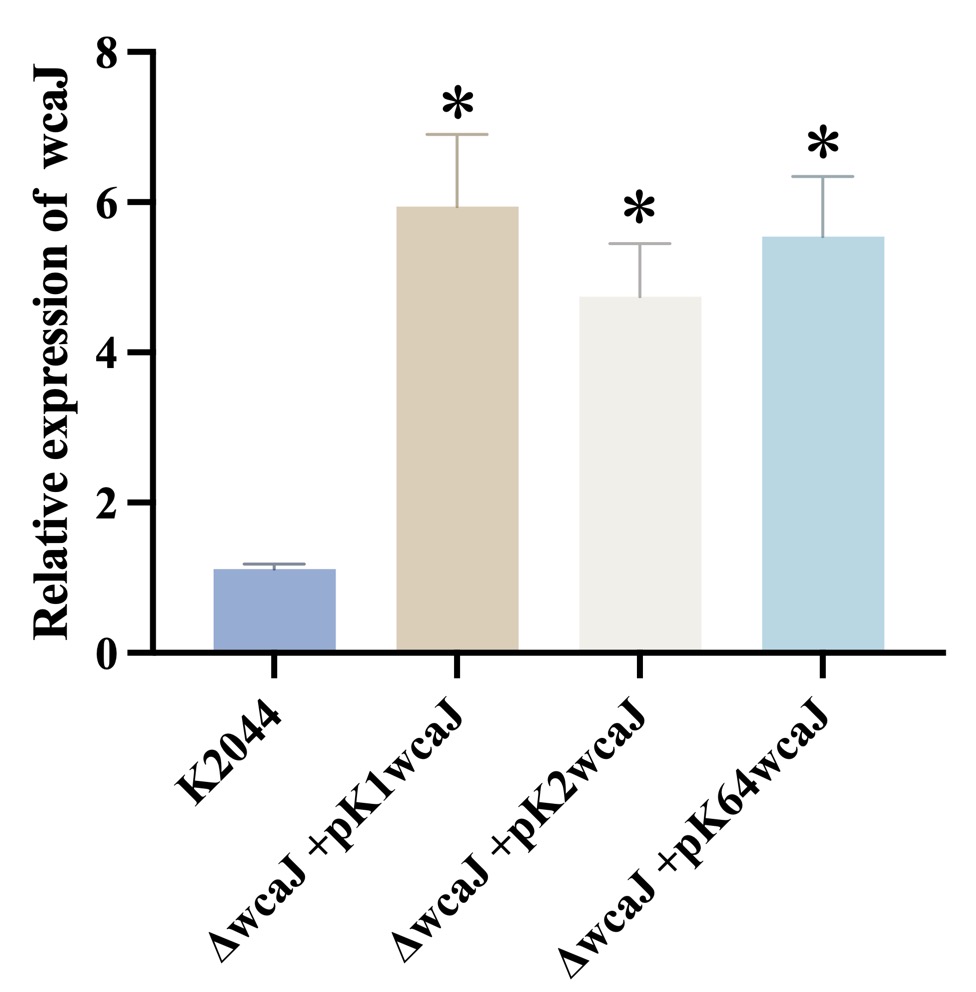

Supplement: Supplementary file 3 [file Image_3.tif]
