# Supplementary material for: Different regulatory mechanisms of the capsule in hypervirulent Klebsiella pneumonia: “direct” wcaJ variation vs. “indirect” rmpA regulation
Source: Front Cell Infect Microbiol. 2023 Apr 25;13:1108818. doi: 10.3389/fcimb.2023.1108818 (PMC10168181; doi:10.3389/fcimb.2023.1108818)
Supplement: Supplementary file 5 [file Table_2.docx]

| **Name** | **Sequences (5’-3’)** | |  |
| --- | --- | --- | --- |
| K1wcaJ-F  K1wcaJ-R  K2wcaJ-F  K2wcaJ-R  K64wcaJ-F  K64wcaJ-R  **ΔwcaJ mutant**  Up-F  Up-R  Kana-F  Kana-R  Down-F  Down-R  **Recombinants**  pK1-F  pK1-R  pK2-F  pK2-R  pK47-F  pK47-R  pK64-F  pK64-R  pACYC184-F  pACYC184-R | | aggtgaaagagttcgaga  gatctgttgcttggacatt  atgtgatgataatgttagcc  taggtgaaaattgccttatag  actgtcgtttgaattctctt  gaactgaactgggcttatac  CTTTGGTAATGTCGATCAGG  atcctgtctcttgatcagatCTGCTTATTGATAAGATGCTGC  ATCTGATCAAGAGACAGGAT  ATGAATCCAGAAAAGCGG  ccgcttttctggattcatCAAGGTTGCCATGAAGATTTC  TGCTGAAAGAAGAGTTTGGAAT  aattgctaacgcagtcaggcGCACAATGCTTATCTTAAGCAGCATC  tgaatccgttagcgaggtgcGGAAATCTTCATGGCAACCTTGTATC  aattgctaacgcagtcaggcAGGGCTAGAAGCTCATAGTGAAATT  tgaatccgttagcgaggtgcTCATGGCAACCTTGTATCATCG  aattgctaacgcagtcaggcATCCCGACTGTTATATAAAGGCTGG  tgaatccgttagcgaggtgcATCTTCATGGCAACCTTGTATCATC  aattgctaacgcagtcaggcATACCCTGCCATGGTTATGGT  tgaatccgttagcgaggtgcTGGCAACCTTGTATCATCGCAT  GCACCTCGCTAACGGATTCA  GCCTGACTGCGTTAGCAATT |  |
