# Supplementary material for: Different regulatory mechanisms of the capsule in hypervirulent Klebsiella pneumonia: “direct” wcaJ variation vs. “indirect” rmpA regulation
Source: Front Cell Infect Microbiol. 2023 Apr 25;13:1108818. doi: 10.3389/fcimb.2023.1108818 (PMC10168181; doi:10.3389/fcimb.2023.1108818)
Supplement: Supplementary file 6 [file Table_3.docx]

| **Name** | **Sequences (5’-3’)** | |  |
| --- | --- | --- | --- |
| qK1wcaJ-F  qK1wcaJ-R  qK2wcaJ-F  qK2wcaJ-R  qK64wcaJ-F  qK64wcaJ-R  rpoB-F  rpoB-R  galF-F  galF-R  wzi-F  wzi-R  manC-F  manC-R | | accaaatgctccaacctctgat  tcaggggtatatgcttcgtca  ctaaccaaatgctccaacctctg  aaggttaagccaggtatcacgg  accaaatactccacccacgg  cctggtattactggtcttgcac  atatgatcaacgccaagccg  atctcagacagcgggttgtt  acctcctacgagctggaagc  gttgtcgccgactaccgg  acagccagcggataactcg  tggttccagaacttcaccgc  atccgaaacagttcctccgc  ccgaccggctcaagaataatgt |  |
