# Supplementary material for: Different regulatory mechanisms of the capsule in hypervirulent Klebsiella pneumonia: “direct” wcaJ variation vs. “indirect” rmpA regulation
Source: Front Cell Infect Microbiol. 2023 Apr 25;13:1108818. doi: 10.3389/fcimb.2023.1108818 (PMC10168181; doi:10.3389/fcimb.2023.1108818)
Supplement: Supplementary file 7 [file Table_4.docx]

Key to supplementary files

Figure S1 The phylogenetic tree of wbaP sequences in hvKp strains.

Figure S2 The mutants of wcaJ sequences in K1 hvKp isolates. (A) nucleotide acid mutants, (B) amino acid mutants.

Figure S3 The expression levels of different wcaJ sequences in K2044K1wcaJ, K2044K2wcaJ, and K2044K64wcaJ strains separately.

Table S1 The characteristics of hvKp in Genbank dataset and clinical isolates.

Table S2 The primes of wcaJ sequences, mutants and plasmids.

Table S3 The primer sequences in qRT-PCR experiments.
